# A phase II study of weekly cisplatin, 6S-stereoisomer leucovorin and fluorouracil as first-line chemotherapy for elderly patients with advanced gastric cancer

**DOI:** 10.1038/sj.bjc.6601280

**Published:** 2003-10-14

**Authors:** F Graziano, D Santini, E Testa, V Catalano, G D Beretta, S Mosconi, G Tonini, V Lai, R Labianca, S Cascinu

**Affiliations:** 1 Medical Oncology Unit, Hospital of Urbino, Italy; 2 Medical Oncology Unit, University Campus Biomedico, Rome, Italy; 3 Medical Oncology Unit, Hospital of Pesaro, Italy; 4 Division of Medical Oncology, Hospital of Bergamo, Italy; 5 Medical Oncology, University of Ancona, Italy

**Keywords:** gastric cancer, chemotherapy, weekly, elderly, phase II

## Abstract

The incidence of gastric cancer (GC) increases significantly after the fifth decade and palliative chemotherapy is the ultimate treatment in the majority of patients. We investigated safety and efficacy of a weekly regimen with cisplatin, fluorouracil and leucovorin as first-line chemotherapy for elderly patients with advanced GC. Chemotherapy-naive patients older than 65 years were considered eligible for study entry. Frail elderly patients were identified and excluded according to the following criteria: age >85 years, dependence in one or more activities of daily living (activities of daily living and instrumental activities of daily living scales), three or more comorbid conditions, one or more geriatric syndromes. Chemotherapy consisted of 1-day per week administration of intravenous cisplatin 35 mg m^−2^, 6S-stereoisomer leucovorin 250 mg m^−2^ and fluorouracil 500 mg m^−2^ (PLF). Patients were re-evaluated after eight weekly cycles and six additional weekly administrations were planned for patients without disease progression. A 5-day subcutaneous filgrastim (5 *μ*g Kg^−1^** **day^−1^, days +1–+5) was used after the first treatment delay for neutropenia and maintained thereafter. In the whole group, the best intention-to-treat overall response rate was 43% (95% CI: 30–56%). The time to disease progression and the median survival time were 5.3 and 8.6 months, respectively. Fatigue was the commonest nonhaematologic toxicity (71% of the patients). Filgrastim was used in 30 patients who showed grade II (20 patients) or grade III (10 patients) neutropenia. Neither grade IV toxicity nor toxic deaths were observed. The weekly PLF regimen resulted safe and effective in elderly patients with advanced GC. This outpatient regimen is based on old and low-cost drugs and it may represent an alternative to new and more expensive combinations.

Gastric cancer is among the most common causes of cancer-related death, and palliative chemotherapy is the ultimate treatment in the majority of patients (Wils, 1998). In recent years, second-generation combination chemotherapy regimens have produced high response rates and impressive survival times (Cascinu *et al*, 1997; Webb *et al*, 1997; Hill and Cunningham, 1998). However, the activity of these treatments has been demonstrated in patients with good performance status and young age and, therefore, they do not reflect the characteristics of patients in the community with advanced gastric cancer (Tebbutt *et al*, 2002). Current epidemiological data show that gastric cancer rarely occurs before the age of 40 years, its incidence increases thereafter and peaks in the seventh decade (Greenlee *et al*, 2000).

Patients older than 65 years have been often excluded from or underrepresented in the study populations of combination chemotherapy trials (Hutchins *et al*, 1999). This demographic selection occurred in the populations of gastric cancer patients treated with intensive combination chemotherapy regimens like the ECF (Tebbutt *et al*, 2002) or the weekly PELF. The weekly PELF (cisplatin, fluorouracil, epi-doxorubicin, leucovorin) regimen plus filgrastim support yielded a 62% response rate and a median survival time of 11 months in a large phase II trial (Cascinu *et al*, 1997). This efficacy was confirmed in subsequent studies (Cascinu *et al*, 1998, 2001), but demographic data suggest that only a minority of patients treated with the weekly PELF regimen were older than 65 years. In the PELF trials, severe haematology and nonhaematologic toxicities were uncommon, but more than half of the patients had mild-to-moderate side effects and even using routine filgrastim support, up to one-third of patients showed grade II or III neutropenia (Cascinu *et al*, 1997, 1998, 2001).

To date, the development of active and safe regimens for treating elderly patients with advanced gastric cancer should be considered a relevant issue in medical oncology. Age itself is not a contraindication to anticancer medical therapies (Goodwin *et al*, 1996; Balducci and Beghe, 2000; Balducci and Yates, 2000) and specific tools are available to identify nonfrail elderly patients who could benefit from chemotherapy (Goodwin *et al*, 1996; Balducci and Yates, 2000).

On these bases, we sought to investigate the weekly combination of cisplatin, fluorouracil and 6S-stereoisomer leucovorin (PLF) as first-line chemotherapy for elderly patients with advanced gastric cancer. Drugs, doses and pace of chemotherapy administrations were those of the weekly PELF regimen, but without epirubicin. This choice was made under the hypothesis that the omission of the anthracycline could have reduced the incidence of neutropenia with improved tolerability of chemotherapy to elderly patients. In a pilot clinical trial, the weekly PLF regimen showed a favourable activity/toxicity ratio and chemotherapy did not require routine filgrastim support (Graziano *et al*, 2002).

## MATERIALS AND METHODS

### Patients characteristics

Chemotherapy-naive patients with pathologically confirmed, relapsed, locally advanced or metastatic gastric cancer were considered eligible for study entry. The study was addressed to patients older than 65 years; frail elderly patients were excluded according to the following criteria: age >85 years, dependence in one or more activities of daily living, presence of three or more comorbid conditions and presence of one or more geriatric syndromes (Balducci and Yates 2000). The Katz and Lawton scales were used to assess activities of daily living (Scott *et al*, 1997); the Katz activities of daily living (ADL) rates the ability to perform routine activities as bathing, dressing, feeding oneself or getting into or out of bed, chairs and vehicles. The Lawton instrumental activities of daily living (IADL) rates more sophisticated functions as the ability to use the telephone, to shop, to handle money, to prepare food or to perform other household tasks. Classic geriatric syndromes which had to be excluded before study inclusion were: dementia, delirium, severe depression, frequent falls, neglect and/or abuse, and spontaneous fractures (Balducci and Yates, 2000). The procedures for performing the above-mentioned geriatric assessments were reported in the study protocol. Each patient was evaluated from the same oncologist at each participating institution.

Additional inclusion criteria were: presence of measurable disease, Eastern Cooperative Oncology Group (ECOG) performance status 0–1, granulocytes count >1500 *μ*l, platelet count >100 000 *μ*l, serum creatinine <1.5 mg dl^−1^ with creatinine clearance ⩾60 ml min^−1^ as estimated by using the Cockcroft–Gault formula (Cockcroft and Gault, 1976), bilirubin level <1.5 mg dl^−1^ and liver enzymes <1.5 times the institutional upper limit. The protocol was approved by each local institutional review board and written informed consent was obtained from all participants.

### Study design

Chemotherapy consisted of a 1-day per week administration of intravenous cisplatin 35 mg m^−2^ with standard hydration and glutathione 1.5 g m^−2^, followed by fluorouracil 500 mg m^−2^ and 6S-stereoisomer leucovorin 250 mg m^−2^ (PLF). All patients received emesis prophylaxis with 5-HT3 inhibitors. Routine prophylactic support with granulocyte colony-stimulating factors was not allowed, but filgrastim was introduced after the first treatment delay for grade II/III neutropenia and maintained thereafter. Erythropoietin was allowed for the treatment of anaemia, when haemoglobin declined to a level ⩽10 mg dl^−1^. Full doses of the anticancer drug were given if granulocyte count was >1500 *μ*l^−1^ and platelet count was >100 000 *μ*l^−1^. In the case of any grade II or more toxicity except alopecia, chemotherapy was delayed a week and then restarted after full recovery. Doses of anticancer drugs were not reduced in the case of grade II–III neutropenia, but these patients started 5-day therapy with filgrastim 5 *μ*g Kg^−1^ day^−1^ (days +1–+5) after the first treatment delay. Similarly, dose of anticancer drugs were not reduced after an episode of grade II–III anaemia and erythropoietin was allowed when haemoglobin levels were ⩽10 mg dl^−1^. Patients with persisting neutropenia or anaemia and who needed a second treatment delay received a 25% dose reduction of all drugs. Also, this dose reduction was planned in the case of grade III toxicity, except alopecia. Patients with unsolved grade II or more toxicity after two consecutive treatment delays, or experiencing grade IV toxicity except alopecia went off study.

### Response and toxicity assessment

Pretreatment evaluation consisted of baseline studies including: medical history, physical examination, blood chemistries, urinoanalysis and ECG. Also, chest X-rays, abdominal computed tomography or magnetic resonance, bone scan and any other test to identify the extent of disease was performed. All patients had physical examination and blood chemistries before each weekly administration of chemotherapy and toxicity was graded according to the National Cancer Institute Common Toxicity Criteria (NCI-CTC, 1998). Tumour measurements for response assessment were performed after eight weekly cycles; patients with responsive or stable disease received six additional weekly cycles and they underwent a second measurement at the end of the treatment program. Follow-up controls were performed every 2 months thereafter. The World Health Organization criteria (WHO, 1979) were used to evaluate responses.

### Statistical plan

The primary end point of this study was to determine the response rate and the toxicity of the weekly PLF regimen in elderly patients with advanced gastric cancer. The secondary objective was to measure the time to disease progression and the survival time.

The study was conducted using the two-stage Simon (1989) design. The sample size was calculated to reject a 10% response rate in favour of a target response rate of 30%, with significance level of 0.05 and a power of 0.90. In the initial stage, a total of 18 evaluable patients were to be entered and evaluated for response. If there were ⩽2 responses, accrual was to be stopped. If >2 responses were observed in the initial stage, then 19 additional patients were to be entered in the second stage to achieve a total of 35 evaluable patients (assuming that two patients might be inevaluable). If >6 responses were observed in 35 patients, an accrual of at least 55 assessable patients was planned to ensure a 25% maximum width of the confidence interval with an expected 30% response rate. The time to disease progression (TTP) was measured from the date of registration to the date of documented disease progression or death. The survival time was measured from the time of registration to the date of death resulting from any cause.

## RESULTS

From January 1999 to December 2001, 58 patients entered on this study. Four patients did not complete eight weekly cycles due to early progression (two patients), refusal after grade III fatigue (one patient) and persistent grade II nephrotoxicity (one patient). The side effects reported by these four patients were included in the overall analysis of toxicity and they were considered as progressions in the intention-to-treat analysis of response. The baseline clinico-pathologic characteristics of the 58 patients are reported in Table 1

**Table 1:** Characteristics of the 58 patients enrolled in the study

Number of patients	58
	
*Sex ratio*	
Male	31
Female	27
Median age (range)	76 years (67–82)
	
*ECOG performance status*	
0	31
I	27
	
*Prior gastrectomy*	
Curative	40
Palliative	12
Not performed	6
	
*Sites of the disease*	
Liver plus lymphnodes	17
Abdominal relapse plus lympnhodes	13
Liver	11
Lymphnodes	10
Primary tumour plus liver	4
Primary tumour plus lympnhodes	2
Lung plus liver	1
	
*CEA level*	
<10 ng ml^−1^	20
>10 ng ml^−1^	38

.

At the end of the treatment program, the best intention-to-treat overall response rate in the 58 patients was 43% (95% CIs: 30–56%) with five complete responses, 20 partial responses, 15 stable diseases and 18 progressions. The five complete responders showed complete disappearance of disease signs after eight weekly administrations and they confirmed the result at the second re-evaluation. The complete responses were obtained in liver metastases (two patients), lymphnodal metastases (two patients), liver plus lymphnodal metastases (one patient). In all, 18 patients achieved partial response after eight weekly cycles and two additional patients with initial stable disease showed partial response after 14 cycles. Three patients with stable disease after eight PLF cycles showed disease progression at the second re-evaluation. In the whole group, the median time to disease progression was 5.3 months and the median survival time was 8.6 months.

Toxicity was generally mild and the maximun grade of haematologic and nonhaematologic toxicities per patient is reported in Table 2

**Table 2:** Treatment-related toxicity in the 58 enrolled patients

	**Number of patients (%)**				
**Adverse event**	**Grade 1**	**Grade 2**	**Grade 3**	**Grade 4**	**All grades**
Neutropenia	10 (17)	20 (35)	10 (17)	0	40 (69)
Thrombocytopenia	8 (14)	2 (3)	0	0	10 (17)
Anaemia	26 (45)	15 (26)	0	0	41 (71)
Nausea	21 (36)	4 (7)	0	0	25 (43)
Vomiting	19 (33)	9 (15)	0	0	28 (48)
Diarrhoea	8 (14)	2 (3)	0	0	10 (17)
Fatigue	18 (31)	22 (38)	1 (2)	0	41 (71)
Anorexia	15 (26)	10 (17)	0	0	25 (43)
Neuropathy	9 (15)	1 (2)	0	0	10 (17)
Nephrotoxicity	4 (7)	1 (2)	0	0	5 (9)
Mucositis	5 (8)	6 (10)	0	0	11 (19)

. According to the treatment protocol, the 30 patients who experienced grade II/III neutropenia started filgrastim which was maintained until the end of the treatment program. The 5-day therapy with filgrastim was started after the fourth PLF administration in 15 patients, after the fifth in nine patients and after the sixth in the remaining six patients.

Fatigue was the commonest treatment-related toxicity and it resulted grade I in 18 patients, grade II in 22 patients and grade III in one patient. Anaemia was recorded in 31 patients whose haemoglobin concentrations declined to a level of 10–12 g dl^−1^ in 26 patients and to 8–9.9 g dl^−1^ in 15 patients. None of the patients showed episodes of grade III anaemia and blood transfusions were not necessary in the study population. Other side effects like stomatitis, peripheral neurotoxicity, nephrotoxicity were uncommon and generally mild. At least one treatment delay was carried out in 45 patients and 22 patients had a 25% dose reduction of anticancer drugs. Neither grade IV toxicity nor toxic death were observed.

## DISCUSSION

Gastric carcinoma often occurs in the 60th and 70th decades, but large trials of palliative chemotherapy in elderly patients are almost lacking. To the best of our knowledge, six phase II studies have been published in international peer-reviewed journals between 1990 and 2002 (Wilke *et al*, 1990; Cascinu *et al*, 1994; Cascinu and Catalano, 1995; Di Bartolomeo *et al*, 1995; Hartung *et al*, 2000; Ikeda *et al*, 2002). In five of these trials, chemotherapy consisted of leucovorin-modulated 5-fluorouracil in combination with cisplatin, epi-doxorubicin, etoposide or mitomycin-C. A phase II study investigated the toxicity profile and the activity of single-agent doxifluridine (Ikeda *et al*, 2002). In general, the tumour control rate seemed promising and no toxic death was observed across all studies. Unfortunately, these trials often enrolled patients with concomitant illnesses or poor performance status and consequently, the number of elderly patients did not exceed 30 in each of them. More recently, Tebbutt *et al* (2002) have reported the results of a phase III trial investigating protracted venous infusion 5-fluorouracil in oesophago-gastric cancer patients. In this study, there was no age limitation for patient eligibility and chemotherapy achieved good results of palliation in a cohort of patients with a median age of 72 years.

In the present investigation, the weekly combination of cisplatin, fluorouracil and folinic acid showed promising tumour control rate and it was safely delivered to nonfrail elderly patients with advanced gastric cancer. Patients were treated on outpatient basis and they showed good compliance to treatment with only one early refusal. A specific assessment of either quality of life or clinical benefit was not planned in this study; however, a record of abdominal pain was performed by means of the memorial pain assessment card and sustained pain relief was observed in 20 out of 28 patients (71%) who were monitored before, during and after PLF chemotherapy.

A major issue in the field of anticancer chemotherapy for elderly patients is the identification of prognostic indicators, which can be used to balance benefits and risks of medical therapies. Many clinical investigations have been addressed to these issues and to date, several prognostic and predictive tools can be used to identify frail and non-frail elderly patients (Reuben, 1997). Also, pharmacokinetic studies have clarified major differences in the metabolism of anticancer drugs in the elderly with specific cares to be used when treating patients ageing more than 65 years (Balducci and Beghe, 1999).

There are many different methods to assess the functional status in elderly patients and the comprehensive geriatric assessment (CGA) is considered as one of the most complete tool for this purpose (Repetto *et al*, 2002). The CGA requires a professional team approach and it may be time consuming and costly (Ingram *et al* 2002). For these reasons, alternative scales and less-extensive methods have been implemented in cancer chemotherapy trials for the evaluation of elderly patients. In particular, the IADL and the ADL scales are considered as the most sensitive assessments of function in older individuals (Balducci and Beghe, 2000), and in both elderly men and women, increasing score on the ADL–IADL scales is predictive of mortality (Scott *et al*, 1997). In the present investigation, the CGA was not included in the study plan, however, specific criteria to exclude frail elderly patients, together with the accurate monitoring of sideeffects and general measures for minimising toxicity allowed the safe and successful administration of combination chemotherapy in a population of patients whose median age was 76 years. Indeed, the eligibility criteria were quite stringent and a proportion of elderly patients could not be enrolled in this trial. On the other hand, we opted for a conservative approach since an unfavourable cost/efficacy ratio may occur when palliative chemotherapy is administered to patients with advanced gastric cancer and low-performance status or comorbities (Janunger *et al*, 2001).

The favourable results of the weekly PLF regimen together with its easiness of administration on outpatient basis and the use of old, low-cost drugs make this treatment worth of further investigation. At some point during chemotherapy, about half of the patients received filgrastim and about 20% of them started erythropoietin. These supports increased treatment-related expenses and, therefore, they may have partially reduced the advantage of low-cost chemotherapeutic agents. In this perspective, further assessments of the weekly PLF regimen could consider a short 3-day filgrastim course between cycles and/or additional treatment delays for neutropenia instead of the immediate use of filgrastim after the first delay. PLF chemotherapy may represent a valid alternative to more expensive combinations including CPT-11 or oxaliplatin and it could be compared to palliative single-agent fluorouracil in a randomised trial for elderly patients with advanced gastric cancer.

## References

[bib1] Balducci L, Beghe C (1999) Pharmacology of chemotherapy in the older cancer patient. Cancer Control 6: 466–470 10758578

[bib2] Balducci L, Beghe C (2000) The application of the principles of geriatrics to the management of the older person with cancer. Crit Rev Oncol Hematol 35: 147–154 10960797 10.1016/s1040-8428(00)00089-5

[bib3] Balducci L, Yates J (2000) General guidelines for management of older patients with cancer. Oncology 14: 221–227 11195414

[bib4] Cascinu G, Catalano G (1995) Intensive weekly chemotherapy for elderly gastric cancer patients using 5-fluorouracil, cisplatin, epi-doxorubicin, 6S-leucovorin and gluthatione with the support of G-CSF. Tumori 81: 32–35 7538704 10.1177/030089169508100107

[bib5] Cascinu S, Fedeli A, Catalano G (1994) Etoposide, leucovorin 5-fluorouracil and interferon alpha-2b in elderly gastric cancer patients: a pilot study. Cancer Chemother Pharmacol 34: 72–74 8174205 10.1007/BF00686115

[bib6] Cascinu S, Graziano F, Barni S, Labianca R, Comella G, Casaretti R, Frontini L, Catalano V, Baldelli AM, Catalano G (2001) A phase II study of sequential chemotherapy with docetaxel after the weekly PELF regimen in advanced gastric cancer. A report from the Italian group for the study of digestive tract cancer. Br J Cancer 84: 470–474 11207039 10.1054/bjoc.2000.1631 PMC2363773

[bib7] Cascinu S, Labianca R, Alessandroni P, Marcellini M, Silva RR, Pancera G, Testa E, Martignoni G, Barni S, Frontini L, Zaniboni A, Luporini G, Cellerino R, Catalano G (1997) Intensive weekly chemotherapy for advanced gastric cancer using fluorouracil, cisplatin, epidoxorubicin, 6S-leucovorin, gluthatione and filgrastim: a report of the Italian Group for the Study of the Digestive Tract Cancer. J Clin Oncol 15: 3313–3319 9363860 10.1200/JCO.1997.15.11.3313

[bib8] Cascinu S, Labianca R, Graziano F, Pancera G, Barni S, Frontini L, Luporini G, Cellerino R, Catalano G (1998) Intensive weekly chemotherapy for locally advanced gastric cancer using 5-fluorouracil, cisplatin, epidoxorubicin, 6S-leucovorin, gluthatione and filgrastim: a report from the Italian Group for the Study of Digestive Tract Cancer (GISCAD). Br J Cancer 78: 390–393 9703289 10.1038/bjc.1998.505 PMC2063041

[bib9] Cockcroft DW, Gault MH (1976) Prediction of creatinine clearance from serum creatinine. Nephron 16: 31–41 1244564 10.1159/000180580

[bib10] Di Bartolomeo M, Bajetta E, de Braud F, Bochicchio AM, Gebbia V, Bozzetti F, Doci R, Bonfanti G, Cozzaglio L (1995) Phase II study of the etoposide, leucovorin and fluorouracil combination for patients with advanced gastric cancer unsuitable for aggressive chemotherapy. Oncology 52: 41–44 7800341 10.1159/000227425

[bib11] Goodwin JS, Samet JM, Hunt WC (1996) Determinants of survival in older cancer patients. J Natl Cancer Inst 88: 1031–1038 8683633 10.1093/jnci/88.15.1031

[bib12] Graziano F, Santini D, Catalano V, Testa E, Tonini G, Lai V, Baldelli AM, Cascinu S (2002) Weekly cisplatin, fluorouracil and folinic acid as first-line chemotherapy for elderly patients with advanced gastric cancer (AGC). Proc Am Soc Clin Oncol 21: 372 (abstr 1484)

[bib13] Greenlee RT, Murray T, Bolden S, Wingo PA (2000) Cancer statistics 2000. CA Cancer J Clin 50: 7–33 10735013 10.3322/canjclin.50.1.7

[bib14] Hartung G, Hofheinz R, Buchheidt D, Rost A, Brecht A, Forche K, Schroder M, Wojatschek C, Fritze D, Hehlmann R, Queisser W. (2000) Combination of bolus 5-fluorouracil, folinic acid and mitomycin-C in advanced gastric cancer: results of a phase II trial. Onkologie 23: 444–447 11441239 10.1159/000027215

[bib15] Hill ME, Cunningham D (1998) Medical management of advanced gastric cancer. Cancer Treat Rev 24: 113–118 9728421 10.1016/s0305-7372(98)90077-9

[bib16] Hutchins LF, Unger JM, Crowley JJ, Coltman Jr CA, Albain KS (1999) Underrepresentation of patients 65 years of age or older in cancer-treatment trials. N Engl J Med 341: 2061–2067 10615079 10.1056/NEJM199912303412706

[bib17] Ikeda N, Shimada Y, Ohtsu A, Boku N, Tsuji Y, Saito H, Koizumi W, Iwase H, Yoshida S, Fukuda H (2002) A phase II study of doxifluridine in elderly patients with advanced gastric cancer: the Japan Clinical Oncology Group study (JCOG 9410). Jpn J Clin Oncol 32: 90–94 11956303 10.1093/jjco/hyf022

[bib18] Ingram SS, Seo PH, Martell RE, Clipp EC, Doyle ME, Montana GS, Cohen HJ (2002) Comprehensive assessment of the elderly cancer patient: the feasibility of self-report methodology. J Clin Oncol 20: 770–7705 11821460 10.1200/JCO.2002.20.3.770

[bib19] Janunger KG, Hafstrom L, Nygren P, Glimelius B, Swedish Council of Technology Assessment in Health Care (2001) A systematic overview of chemotherapy effects in gastric cancer. Acta Oncol 40: 309–326 11441938 10.1080/02841860151116385

[bib20] National Cancer Institute Common Toxicity Criteria (1998), Version 2.0. January 30, 1998. http://ctep.info.nih.gov/CTC3/ctc.htm.0 15332541

[bib21] Repetto L, Fratino L, Audisio RA, Venturino A, Gianni W, Vercelli M, Parodi S, Dal Lago D, Gioia F, Monfardini S, Aapro MS, Serraino D, Zagonel V (2002) Comprehensive geriatric assessment adds information to Eastern Cooperative Oncology Group performance status in elderly patients: an Italian Group for Geriatric Oncology Study. J Clin Oncol 20: 494–502 11786579 10.1200/JCO.2002.20.2.494

[bib22] Reuben DB (1997) Geriatric assessment in oncology. Cancer 80: 1311–1316 9317184 10.1002/(sici)1097-0142(19971001)80:7<1311::aid-cncr17>3.0.co;2-a

[bib23] Scott WK, Macera CA, Cornman CB, Sharpe PA (1997) Functional health status as a predictor of mortality in men and women over 65. J Clin Epidemiol 50: 291–296 9120528 10.1016/s0895-4356(96)00365-4

[bib24] Simon R (1989) Optimal two-stage designes for phase II clinical trials. Controlled Clin Trials 10: 1–10 2702835 10.1016/0197-2456(89)90015-9

[bib25] Tebbutt NC, Norman A, Cunningham D, Iveson T, Seymour M, Hickish T, Harper P, Maisey N, Mochlinski K, Prior Y, Hill M (2002) A multicentre, randomized phase III trial comparing protracted venous infusion (PVI) 5-fluorouracil (5-FU) with PVI 5-FU plus mitomycin C in patients with inoperable oesophago-gastric cancer. Ann Oncol 13: 1568–1575 12377644 10.1093/annonc/mdf273

[bib26] Wilke H, Preusser P, Fink U, Achterrath W, Lenaz L, Stahl M, Schober C, Link H, Meyer HJ, Lucke B (1990) High dose folinic acid/etoposide/5-fluorouracil in advanced gastric cancer: a phase II study in elderly patients or patients with cardiac risk. Invest New Drugs 8: 65–70 2345071 10.1007/BF00216926

[bib27] Wils J (1998) Treatment of gastric cancer. Curr Opin Oncol 10: 357–361 9702404 10.1097/00001622-199807000-00013

[bib28] Webb A, Cunningham D, Scarffe JH, Harper P, Norman A, Joffe JK, Hughes M, Mansi J, Findlay M, Hill A, Oates J, Nicolson M, Hickish T, O'Brien M, Iveson T, Watson M, Underhill C, Wardley A, Meehan M (1997) Randomized trial comparing epirubicin, cisplatin, and fluorouracil versus fluorouracil, doxorubicin, and methotrexate in advanced esophagogastric cancer. J Clin Oncol 15: 261–267 8996151 10.1200/JCO.1997.15.1.261

[bib29] World Health Organization (1979) Handbook for reporting results of cancer treatment. Geneva: World Health Organization

